# The surge of mpox in African countries

**DOI:** 10.11604/pamj.supp.2025.50.1.46935

**Published:** 2025-03-02

**Authors:** Abdou Salam Gueye

**Affiliations:** 1 World Health Organization, Regional Office for Africa, Emergency Preparedness and Response Programme, Brazzaville, Congo Republic

**Keywords:** Monkeypox, zoonosis, Africa

## Editorial


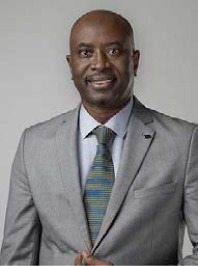


Mpox, formerly known as Monkeypox, is a zoonotic disease caused by the Monkeypox virus, belonging to the Orthopox virus genus within the Poxviridae family. Although the virus was first isolated in Denmark in 1958 in a colony of captive monkeys who developed smallpox-like illnesses, it was not until 1970 that human cases were documented [1-3]. The main animal hosts of Monkeypox virus (MPXV) include African rodents, prairie dogs, mice, and some species of monkeys [1-5]. It remained under relative neglect by the global community until the first global outbreak in 2022, which prompted the World Health Organization to declare mpox a Public Health Emergency of International Concern (PHEIC) in July 2022, 5, 6. As of 22 December 2023, close to 93,000 laboratory-confirmed mpox cases and 171 deaths have been reported from 116 countries, including 2,126 cases and 22 deaths from endemic regions of Africa.

In Africa, transmission is mainly from a zoonotic source through contact with blood, body fluids, or cutaneous or mucosal lesions of infected animals, especially rodents and primates [6-9]. With the disease largely confined to the rural areas of Central and West Africa for decades, the reason for which it did not command broad attention or concern [9]. The global health community overlooked the potential for broader threats associated with the virus and instead focused on more immediate and obvious global health threats [5].

However, on August 14, 2024, the WHO declared its second PHEIC, following new realizations and reprioritization of mpox as a neglected tropical disease with pandemic potential [5,9-11]. Earlier, on August 13, 2024, the Africa Centre for Disease Control (Africa CDC) declared mpox a Public Health Emergency of Continental Security (PHECS). These declarations underscored the importance of including the disease in pandemic prevention, preparedness, and response plans. They are important primarily to drive the containment of the outbreaks in Africa and secondarily to alert countries to intensify their reporting of cases and prevention programs. The declarations also constituted calls to stop the neglect as well as for global solidarity and for high-income countries to support low-income countries to control the outbreaks [9].

While outbreaks outside of Africa were swiftly controlled, the situation in Africa, particularly in the DRC, continues to raise concerns. From 1st January 2022 to November 2023, DRC reported 19,034 suspected cases and 820 deaths, of which 13,357 cases and 607 deaths were reported from January to December 2023 alone. Of particular concern is the significant feature of sexual transmission in the DRC, indicating a pressing need for comprehensive in-country and regional response efforts.

The 2024 DRC outbreak and concerns for regional mpox containment in endemic African countries prompted the organization of an emergency regional meeting by the WHO, Africa CDC, Institut National de Recherche Biomédicale (INRB), and UNICEF. The meeting was held in Kinshasa DRC from April 11th to 13th, 2024, and convened stakeholders from endemic and at-risk countries, multilateral and bilateral partners, funding agencies, and research institutions to assess the current outbreak and devise a regional response strategy. Among its outcomes, the meeting formulated a strategic approach to address significant knowledge gaps essential for informing effective mpox response efforts. Shortly after this meeting the outbreak spread to other countries and became a continent-wide concern.

There has been a significant surge in the number of confirmed cases and deaths in 2024 compared to the same period in 2023. As of October 2024, Africa reported 50,840 mpox cases, including 10,741 cases confirmed through real-time PCR and 1083 deaths in 14 countries, a 545% rise in confirmed cases from 2023 [5,10]. Furthermore, this current outbreak is occurring simultaneously across multiple countries, including new health districts where the disease had not been previously reported [5]. However, the majority of cases so far are in the Democratic Republic of Congo [11]. In addition, many of the confirmed cases of mpox in this current outbreak are a result of the newly identified clade 1b, which seems to be easily transmitted from humans to humans through sexual contact among other modes [12].

The central African region accounts for 85.8% of cases and 99.4% of deaths. By the 100 days of the outbreak, 14 countries still reported new cases, although October 2024, saw a 25% decline. Children younger than 14 years account for 38.9% of cases, mostly in the Democratic Republic of the Congo, Burundi, Nigeria, Côte d’Ivoire, and Central African Republic. South Africa, Kenya, and Uganda link mortality to unmanaged HIV. Surveillance remains weak, with only 3% of contacts traced and laboratory testing rates at 47% [13]. By February 13, 2025, 27 new laboratory-confirmed cases were reported from Nigeria (25 retrospectively), Liberia (1), and Kenya (1) with 1 death from Nigeria. Thirteen countries are assessed as active: DRC, Burundi, CAR, Côte d’Ivoire, Liberia, Nigeria, Rwanda, Uganda, Kenya, Republic of Congo, Zambia, Sierra Leone and South Sudan. Cameroon, Ghana, Angola, and Guinea are in the control phase while South Africa, Gabon, Zimbabwe, and Mauritius are at the end of the outbreak with no new cases for more than 90 days.

Gabon, Zimbabwe, and Mauritius are at the end of the outbreak with no new cases for more than 90 days. Peer-reviewed literature on mpox in Africa, particularly articles published in African journals, remains scarce. To bridge this knowledge gap, the WHO Health Emergencies Programme (WHE), in collaboration with leading African experts on mpox, proudly presents this special issue to enrich the understanding of mpox in endemic countries of Africa. The special issue received submissions related to all aspects of mpox in Africa, including epidemiology, research, clinical practice, outbreak investigation and response, and other relevant mpox data and activities in Africa. Submissions focused on mpox research conducted outside Africa will be considered if deemed relevant to outbreak response efforts in endemic African countries. Except for editorials, all submissions underwent rigorous external peer review. I have the pleasure of recommending this to all.
